# MRCT and CT in the diagnosis of pediatric disease imaging: assessing imaging performance and clinical effects

**DOI:** 10.1186/s12880-024-01273-w

**Published:** 2024-04-25

**Authors:** Xiaofei Wang, Wen Hu

**Affiliations:** Department of Radiology, Xi’an Children’s Hospital, No. 69, Xijuyuan Lane, Lianhu District, 710003 Xi’an, Shaanxi Province China

**Keywords:** Magnetic resonance imaging, Computed tomography, Cerebral palsy, Imaging performance, Clinical results, Encephalomalacia, Cerebral ischemia, Gray-white matter atrophy of the brain

## Abstract

**Objective:**

This study focused on analyzing the clinical value and effect of magnetic resonance imaging plus computed tomography (MRCT) and CT in the clinical diagnosis of cerebral palsy in children.

**Methods:**

From February 2021 to April 2023, 94 children diagnosed with cerebral palsy were selected from our hospital for study subjects. These patients were divided into CT and MRI groups, with CT examination given to the CT group and MRI examination given to the MRI group. The positive rate of the two examination methods in the diagnosis of cerebral palsy was compared, different imaging signs in two groups of children with cerebral palsy were compared, and the diagnostic test typing results between two groups were further analyzed.

**Results:**

The diagnostic positivity rate of the children in the MRI group was 91.49%, which was significantly higher than that of the children in the CT group (70.21%) (*P* < 0.05). In both groups, encephalomalacia, bilateral frontal subdural effusions, and gray-white matter atrophy of the brain were the main signs, and the difference in the proportion of these three imaging signs between the two groups was not significant (*P* > 0.05). Differences between the two groups examined for cerebral palsy subtypes were not significant (*P* > 0.05).

**Conclusion:**

The positive rate of pediatric cerebral palsy examined by MRI is higher than that of CT diagnosis, but the clinic should organically combine the two to further improve the detection validity and accuracy.

## Introduction

Cerebral palsy describes the most common physical disability in childhood [1]. Cerebral palsy possesses multiple etiologies leading to brain injury that can affect movement, posture, and balance. The movement disorders that have an association with cerebral palsy can be categorized as spasticity, dyskinesia, ataxia, mixed and others [2]. Cerebral palsy is a developmental disorder of movement and posture, and early identification is important in cerebral palsy management, which allows early access to cerebral palsy interventions [3]. Early diagnosis is involved with the use of neuroimaging, and standardized neurological and motor assessments that can indicate congruent abnormal findings revealing cerebral palsy [4]. Cerebral palsy diagnosis is on the basis of a history of abnormal motor development and an examination “placing” lesions in the brain [5].

Neuroimaging, especially with magnetic resonance (MR) techniques, has the ability to provide insights into the pattern and severity of cerebral injuries underlying cerebral palsy [6]. Neuroimaging is reported to strengthen the relation of patterns in the developing brain with cerebral palsy. Not only computed tomography (CT) but also magnetic resonance imaging (MRI) can identify developmental malformations at an early stage [7]. MRI is one of preventive and diagnostic techniques for cerebral palsy [8]. MRI plays a considerable role in the diagnosis of movement disorders, and the combination of clinical features with MRI and single-photon emission CT is recommended for optimize the diagnostic algorithm in movement disorders [9]. MRI is able to demonstrate and differentiate a variety of insults and anomalies that are responsible for cerebral palsy [10]. A previous study has reported that cerebral palsy is mainly featured with brain lesions that can be identified by MRI in around 75% of preterm infants [11]. MRI is a recommended technique in cerebral palsy children where the main presenting problem in cerebral palsy is decreased motor ability and the aetiology has not been established [12]. MRI is useful in the understanding and evaluation of cerebral palsy [13]. MRI scans can help reveal cerebral palsy pathological basis and strongly correlate with clinical results [14]. Cerebral palsy clinical spectrum is variable, and CT scan of brain is a significant mode of diagnosis and prognosis [15]. However, the clinical value of MRI & CT (MRCT) in cerebral palsy in children is rarely discussed. Therefore, this study was aimed at probing the clinical value and effect of MRCT and CT in the clinical diagnosis of cerebral palsy in children.

## Materials and methods

### Ethics statement

The study was under the approval of the Ethic Committee of Xi’an Children’s Hospital and conformed to Helsinki Declaration. The children’s families or guardians agreed and signed the informed consent form.

### General data

The subjects of this study were 94 children with cerebral palsy admitted to Xi’an Children’s Hospital from June 2021 to April 2023. They were separated into 2 groups based on the imaging diagnostic methods actually received by the children, those who underwent CT examination were set as the CT group, and those who underwent MRI examination were set as the MRI group, with 47 cases in each group. In the MRI group, there were 30 males and 17 females, with an average age of 2.64 ± 1.15 years. In the CT group, there were 35 males and 12 females, with a mean age of 2.68 ± 1.37 years old. No difference was found in the general data of children in the two groups (*P* > 0.05), which was comparable.

Inclusion criteria: patients meeting the diagnostic criteria for cerebral palsy in children [16, 17]; those aged 3 months-5 years, those with complete imaging data such as CT and MRI examination.

Exclusion criteria: those combined with psychiatric or cognitive disorders; those with severe primary diseases of heart, liver, kidneys and other major organs; those with limb movement disorders and progressive paralysis due to other diseases, and those with metabolic diseases.

### Test methods

In the CT group, brain CT examination was performed. Before the examination, it is necessary to ensure that the child is in a natural state of sleep, if not, then follow the doctor’s instructions to give 10% chloral hydrate solution for sedation, and the examination will be carried out after the child is asleep. Siemens SOMATOM Drive dual source CT was applied, and children were put in supine position, and routine craniocerebral axial plain scanning was performed with 130 mA, 120 kV, layer thickness and layer spacing of 10 mm, and scanning time at 0.5 s/circle.

**Fig. 1:**
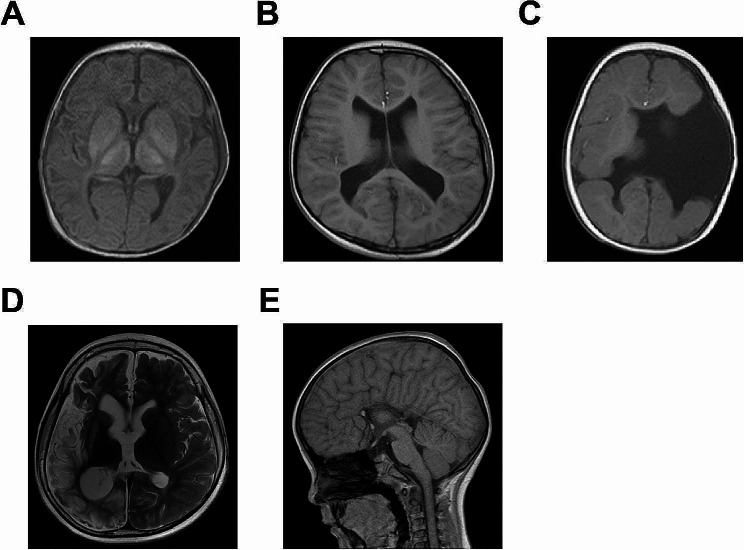
Representative MRI images of children with cerebral palsy. (**A**) Image of a child with cerebral palsy with cerebral ischemia. (**B**) Image of a child with cerebral palsy with encephalomalacia. (**C**) Image of a child with cerebral palsy with schizencephaly. (**D**) Image of a child with cerebral palsy with cerebral atrophy. (**E**) Image of a child with cerebral palsy with agenesis of the corpus callosum

In the MRI group, the children were subjected to MRI examination using a Siemens 1.5T Avanto magnetic resonance instrument with an 8-channel combined head and neck coil, and the subject’s head was immobilized. Children who could not cooperate were sedated with 10% chloral hydrate solution at a dose of 0.5 ml/kg, and scanned after they were asleep. All subjects had cotton balls of appropriate size inserted in their ears to minimize the effect of noise. All subjects first underwent routine T1WI, T2WI and FLAIR scans. The scanning routines for T1WI, T2WI and FLAIR were as follows: T1WI scanning parameters were TR/TE 2175/16 ms, layer thickness 6 mm, layer spacing 2 mm, matrix 320 × 192, and FOV 240 × 240 mm; T2WI scanning parameters were TR/TE 4000/102 ms, layer thickness 6 mm, layer spacing 2 mm, matrix 384 × 256, and FOV 240 × 240 mm; FLAIR scanning parameters were TR/TE 9000/117 ms, layer thickness 6 mm, layer spacing 2 mm, matrix 256 × 224, and FOV 240 × 240 mm. DTI scanning was then performed, choosing a transverse position, using a single excitation SE EPI sequence with scanning parameters of TR/TE 9200/79.6 ms, matrix 96 × 96, Phase FOV 1.0, b = 800 s/mm^2^, number of dispersion-sensitive gradient directions of 17, layer thickness of 3.5 mm, and layer spacing of zero. The resulting data was transferred to a workstation and analyzed using DTI processing software. At the early stage of examination and after the examination, a comprehensive nursing intervention was carried out, which mainly included visual and auditory stimulation to the children, and passive or voluntary movement of the children. Representative MRI images of children with cerebral palsy are shown in Fig. 1.

### Observation indicators

After the above diagnosis, the diagnosis results were made by two experienced clinicians, and consensus was reached when different diagnostic results existed. The positive rate of the two test modalities for cerebral palsy diagnosis was compared, and the diagnostic accuracy of the two test methods for different imaging signs and different clinical typings of cerebral palsy children was compared.

### Statistics

All data were statistically analyzed implementing SPSS 26.0 (IBM SPSS Statistics, Chicago, IL, USA). Measurement data were described by mean ± standard deviation, and inter-group comparisons were analyzed using Student’s t-test. Numeration data were expressed as n (number of cases) and % (percentage), and the between-group comparisons were performed using the chi-square test. *P* < 0.05 was considered a statistically significant difference.

## Results

### Comparison of positive diagnostic rates for cerebral palsy between two groups of children

In the MRI group, a total of 43 children were diagnosed with cerebral palsy by MRI, with a positive rate of 91.49%, while in the CT group, 33 cases of children were diagnosed with cerebral palsy, with a positive rate of 70.21%. After analyzing the results by chi-square test, the results showed that the positive rate of the MRI group was higher versus the CT group, and the difference was statistically significant (*P* < 0.05) (Table 1).

**Table 1:** Comparison of positive diagnostic rates for cerebral palsy between two groups of children [n (%)]

Group	Encephalomalacia	Cerebral ischemia	Brain dysplasia	Hydrocephalus	Meningitis	White matter abnormality	Positive rate
CT group (*n* = 47)	9 (19.15)	0 (0.00)	5 (10.64)	10 (21.28)	2 (4.26)	7 (14.89)	33 (70.21)
MRI group (*n* = 47)	11 (23.40)	2 (4.26)	6 (12.77)	12 (25.53)	3 (6.38)	9 (19.15)	43 (91.49)
χ^2^							6.871
*P*							0.009

### Different imaging signs in two groups of children with cerebral palsy

The main causes of the disease in MRI and CT groups could be categorized as birth injury, congenital dysplasia, and cerebral hypoxia and ischemia. Among the 33 children with positive CT and 43 children with positive MRI, the main imaging signs in children with birth injury (5 cases) were meningitis (3 cases) and encephalitis (2 cases), and in children with congenital dysplasia (24 cases), the main imaging signs were multiple calcified plaques in the brain (2 cases), encephalomalacia (12 cases), anencephalic malformation (1 case), and megalencephalic malformation (1 case), gray-white matter displacement of the brain (2 cases), schizencephaly (2 cases), agenesis of the corpus callosum (3 cases), and cerebral connectivity malformation (1 case); and in children with hypoxic-ischemic etiology (47 cases), the main imaging signs were cerebral ischemic lesions (3 cases), white-matter degeneration of the brain (3 cases), hydrocephalus (7 cases), gray-white matter atrophy of the brain (11 cases), encephalomalacia (8 cases), and bilateral frontal subdural effusions (15 cases). The above imaging signs were ranked according to the number of cases, and the top three types of imaging signs in the children who received CT examination and those who received MRI examination were encephalomalacia, bilateral frontal subdural effusions, and gray-white matter atrophy of the brain. The chi-square test analysis demonstrated that the comparison of the proportions of the top three imaging signs between the two groups of children was not significant (*P* > 0.05). It was also found that mild white-matter degeneration of the brain and ischemic lesions were slightly more common in children who underwent MRI than in those who underwent CT, and multiple calcified plaques in the brain were slightly more common in children who underwent CT than in those who underwent MRI (Table 2).

**Table 2:** Different imaging signs in two groups of children with cerebral palsy (n/%)

Group	CT group (*n* = 47)	MRI group (*n* = 47)	*P*
Encephalomalacia	9 (19.15)	11 (23.40)	0.614
Bilateral frontal subdural effusions	7 (14.89)	8 (17.02)	0.778
Gray-white matter atrophy of the brain	6 (12.76)	5 (10.64)	0.748
Cerebral ischemic lesions	0 (0.00)	3 (6.38)	0.242
White matter degeneration of the brain	0 (0.00)	3 (6.38)	0.242
Multiple calcified plaques in the brain	2 (4.26)	0 (0.00)	0.495
Anencephalic or megalencephalic malformations	1 (2.13)	1 (2.13)	> 0.999
Agenesis of the corpus callosum	1 (2.13)	2 (4.26)	0.557
Schizencephaly	1 (2.13)	1 (2.13)	> 0.999
Cerebral connectivity malformation	0 (0.00)	1 (2.13)	> 0.999
Gray-white matter displacement of the brain	1 (2.13)	1 (2.13)	> 0.999
Hydrocephalus	3 (6.38)	4 (8.51)	0.694
Meningitis and encephalitis	2 (4.26)	3 (6.38)	0.646

### Comparison of diagnostic test typing results between two groups

Of the 33 children with positive CT findings and 43 children with positive MRI findings, 59 were spastic, 1 dyskinetic, 0 ataxic, 11 hypotonic, and 5 mixed. In detail, 26 were spastic, 1 dyskinetic, 0 ataxic, 4 hypotonic, and 2 mixed in the CT group; 33 were spastic, 0 dyskinetic, 0 ataxic, 7 hypotonic, and 3 mixed in the MRI group. Analysis by chi-square test showed that there was no significant difference between the cerebral palsy subtypes results of the two groups (*P* > 0.05) (Table 3).

**Table 3:** Comparison of diagnostic test typing results between two groups

Clinical typing	CT group (*n* = 47)	MRI group (*n* = 47)	*P*
Spastic type	26(55.32)	33 (70.21)	0.135
Dyskinetic type	1 (2.13)	0 (0.00)	> 0.999
Ataxia type	0 (0.00)	0 (0.00)	> 0.999
Hypotonic type	4 (8.51)	7 (14.89)	0.336
Mixed type	2 (4.26)	3 (6.38)	0.646

## Discussion

Cerebral palsy is the most common chronic disability in childhood [18] that can affect musculoskeletal development [19]. This study focused on the clinical value and effect of MRCT and CT in the clinical diagnosis of cerebral palsy in children.

As previously reported, MRI is more recommended to utilized combined with neuroimaging in contrast with CT [5]. MRI benefits in high-risk term infants [20]. CT plays a role in emergency department patients with suspected sepsis and CT shows a higher positive predictive value with regard to the discharge diagnosis [21]. A movement toward more judicious use of CT imaging emerges in an attempt to reduce exposure of pediatric patients to ionizing radiation [22]. Therefore, our paper focused on the role of MRCT in the diagnosis of pediatric diseases. We compared the positive diagnostic rates for cerebral palsy between two groups of children, and found that the positive rate of the MRI group was higher versus that of the CT group. In detail, in the MRI group, a total of 43 children were diagnosed with cerebral palsy by MRI, with a positive rate of 91.49%, while in the CT group, 33 cases of children were diagnosed with cerebral palsy, with a positive rate of 70.21%. The results of the study demonstrates that in making the diagnosis of pediatric cerebral palsy, the MRI examination is more advantageous than CT examination, which is in general agreement with most of the previous statistical results in the literature.

MRI is implemented to identify intracranial abnormalities and it possesses greater diagnostic accuracy in preterm infants [23]. In a previous study, MRI has shown the best accuracy of 84% [24]. Brain CT represent high diagnostic performance in the detection of facial bone fractures with high accuracy in pediatric patients [25]. In our study, we compared different imaging signs in two groups of children with cerebral palsy and found that the top three types of imaging signs in the children who received CT examination and those who received MRI examination were encephalomalacia, bilateral frontal subdural effusions, and gray-white matter atrophy of the brain. In addition, mild white-matter degeneration of the brain and ischemic lesions were slightly more common in children who underwent MRI than in those who underwent CT. The results showed that the diagnosis rates of CT and MRI were comparable for the more common imaging signs such as encephalomalacia, bilateral frontal subdural effusions, and gray-white matter atrophy of the brain. However, for white-matter degeneration of the brain and ischemic lesions, the diagnosis rate of MRI examination was higher than that of CT examination. In other words, for the diagnosis of white-matter degeneration of the brain and ischemic lesions, the diagnosis rate of MRI examination was superior. Besides, multiple calcified plaques in the brain were slightly more common in children who underwent CT than in those who underwent MRI, this suggests the significant advantage of CT in the diagnosis of this sign, which can effectively solve the problem of MRI’s difficulty in detecting small multiple calcified plaques in the brain due to technical factors. For this reason, we recommend that, in practice, the two tests should be used in combination in cases where the diagnosis cannot be confirmed, so as to ensure that the diagnosis can be confirmed in the first instance, thus buying more and more valuable time for further treatment. A previous study has demonstrated that the findings of MRI for patients with spastic cerebral palsy have a close relation to cerebral palsy types [13]. Furthermore, we also compared diagnostic test typing results between two groups, and it was found that of the 33 children with positive CT findings and 43 children with positive MRI findings, 59 were spastic, 1 dyskinetic, 0 ataxic, 11 hypotonic, and 5 mixed. In detail, 26 were spastic, 1 dyskinetic, 0 ataxic, 4 hypotonic, and 2 mixed in the CT group; 33 were spastic, 0 dyskinetic, 0 ataxic, 7 hypotonic, and 3 mixed in the MRI group. Analysis by chi-square test showed that there was no significant difference between the cerebral palsy subtypes results of the two groups.

In summary, this research demonstrates that the positive rate of pediatric cerebral palsy examined by MRI is higher than that of CT diagnosis, but the clinic should organically combine the two to further improve the detection validity and accuracy. This study lays a foundation to explore the effects and clinical value of MRCT in children with cerebral palsy. Nevertheless, further exploration is necessary to further convince our findings.

## Data Availability

The experimental data used to support the findings of this study are available from the corresponding author upon request.
